# *Streptococcus agalactiae* strain COH1 transcriptome in association with stem cell-derived brain-like endothelial cells

**DOI:** 10.1128/mra.00455-24

**Published:** 2024-11-11

**Authors:** Nadine Vollmuth, Till Sauerwein, Konrad U. Foerstner, Alexander J. Westermann, Alexandra Schubert-Unkmeir, Daryl W. Lam, Brandon J. Kim

**Affiliations:** 1 Department of Biological Sciences, University of Alabama, Tuscaloosa, Alabama, USA; 2 Institute of Molecular Infection Biology (IMIB), University of Wuerzburg, Wuerzburg, Germany; 3 ZB MED, Information Centre for Life Sciences, Cologne, Germany; 4 TH Koeln, University of Applied Sciences, Cologne, Germany; 5 Helmholtz Institute for RNA-based Infection Research (HIRI), Helmholtz Centre for Infection Research (HZI), Wuerzburg, Germany; 6 Institute for Hygiene and Microbiology, University of Wuerzburg, Wuerzburg, Germany; 7 Department of Microbiology, University of Alabama at Birmingham Heesink School of Medicine, Birmingham, Alabama, USA; 8 University of Alabama Center of Convergent Biosciences and Medicine, Tuscaloosa, Alabama, USA; 9 University of Alabama Life Research, Tuscaloosa, Alabama, USA; Rochester Institute of Technology, Rochester, New York, USA

**Keywords:** Group B *Streptococcus*, transcriptome, blood-brain barrier

## Abstract

*Streptococcus agalactiae* (Group *B Streptococcus*) strain COH1 is a representative strain of serotype III, multi-locus sequence type 17, which is disproportionately associated with neonatal meningitis. Here we report the transcriptome of COH1 when interacting with human brain endothelial cells compared with COH1 alone.

## ANNOUNCEMENT

To cause bacterial meningitis, bacteria must interact with and penetrate the blood-brain barrier (BBB) (1). The BBB is composed of highly specialized brain endothelial cells (BECs) that serve to maintain barrier integrity and prevent pathogens from exiting the circulation and entering the central nervous system (2). The surface of BECs represents an environment that meningitis-causing bacteria must interact with. *Streptococcus agalactiae* (Group B *Streptococcus* [GBS]) is the leading cause of neonatal meningitis, and presently, there is no vaccine available (1, 3, 4). GBS serotype III, multi-locus sequence type (MLST) 17, is disproportionately associated with neonatal meningitis (1, 5, 6). Much work has been conducted to identify how the BBB responds to bacteria such as GBS (1, 7–11). However, little is known about how GBS responds to association with BECs.

**Fig 1:**
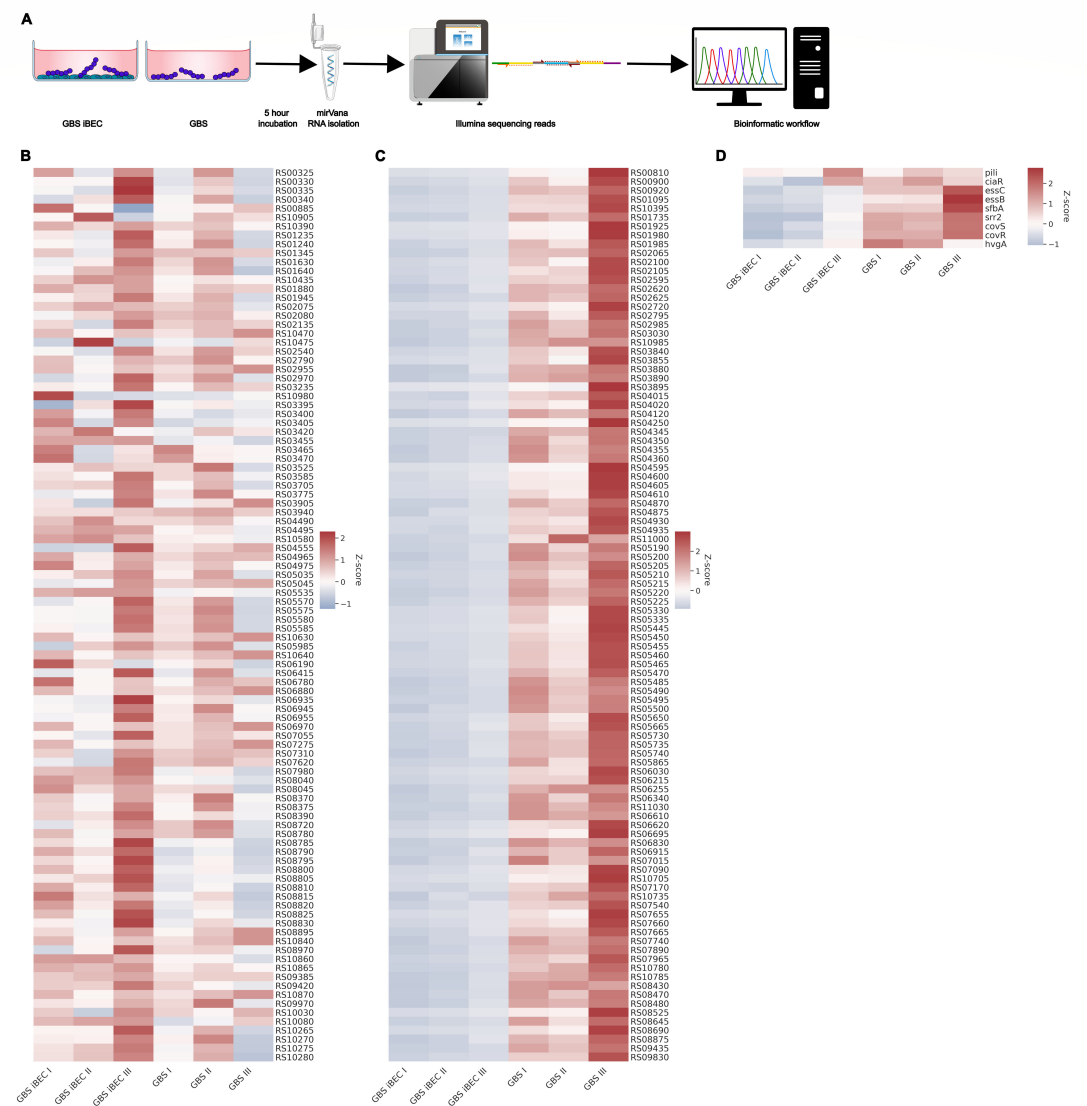
Heatmaps of COH1 transcriptome when interacting with human brain endothelial cells compared with COH1 alone. (**A**) Schematic of the workflow: Group B *Streptococcus* (GBS) and induced pluripotent stem cell-derived brain-like endothelial cells (iBECs). (**B–D**) Heatmap of differentially regulated genes: (**B**) top upregulated genes, (**C**) top downregulated genes, and (**D**) selection of known GBS adhesins.

GBS strain COH1 (serotype III, MLST-17, a clinical isolate obtained from a newborn) was either incubated alone in wells containing endothelial cell (EC) media (hESFM + B27) or in wells containing EC media and human induced pluripotent stem cell-derived brain-like endothelial cells (iBECs) for 5 h at 37°C + 5% CO_2_ (multiplicity of infection of 10) (10, 12–17). In the publication originating the data set presented here, more details are available on the methods for iBEC obtention (18). After washing away non-cell-associated bacteria with phosphate-buffered saline, total RNA was immediately collected using the mirVana RNA (Thermo Scientific) isolation kit (Fig. 1A) (18). This process was repeated three times to collect individual biological replicates. The quality of the extracted total RNA samples was checked using the Agilent RNA 6000 Nano Kit, assay mode Prokaryote Total RNA Nano, on a Bio Analyzer. cDNA libraries were prepared as previously described (19). For cDNA library preparation, the Illumina Stranded Total RNA Prep Ligation with Ribo-Zero Plus protocol was used according to the manufacturer’s recommendations. Libraries were pooled in equimolar concentrations and fractionated using the Agencourt AMPure kit and sequenced on an Illumina NextSeq500 platform in single-end mode for 75 cycles. Obtained reads (Illumina read length 75 bp) were trimmed using Cutadapt (version 1.15), and Illumina TruSeq adapters were removed from the 3′ end. Reads with a Phred quality score less than 20 and following downstream bases were cut. READemption (version 0.4.3, DOI: 10.5281/zenodo.250598) was used for downstream mapping (segemehl version 0.2.0 associated with READemption) and analysis (20–23). *Streptococcus agalactiae* COH1 genome and annotation from National Center for Biotechnology Information GenBank (accession number HG939456.1, assembly accession number GCA_000689235.1) were extended with an existing sRNA annotation (24). Differential gene expression was performed with R package DESeq2 (version 1.18.1, R version 3.4.4) based on raw read counts (25). As differentially expressed, were genes defined with an adjusted (Benjamini-Hochberg-corrected) *P* value of 0.05. The complete bioinformatic workflow and number of reads of the individual conditions and replicates can be found under DOI https://zenodo.org/records/12652566. These methods are expanded versions of descriptions in our related work (18).

Compared with GBS in media alone, 430 of the 2,068 total annotated genes were found to be differentially regulated in GBS associated with iBECs, with 360 genes exhibiting downregulation (18). These data suggest that GBS can sense host cell association and accordingly adapts its gene expression pattern (Fig. 1B and C). Examination of known GBS adhesins that have been demonstrated to facilitate interaction with BECs does not show differential regulation at the transcript level, suggesting that these factors are not upregulated when interacting with the BBB (Fig. 1D) (6, 7, 26–28). Our data suggest that other contributing factors may facilitate GBS intimate interaction with BECs, and presently unknown mechanisms of BBB dysfunction could be discovered by examining the differentially expressed bacterial genes uncovered here (18).

## Data Availability

Data have been deposited in the National Center for Biotechnology Information’s Gene Expression Omnibus under accession number GSE197489.
